# Highlighting the DNA damage response with ultrashort laser pulses in the near infrared and kinetic modeling

**DOI:** 10.3389/fgene.2013.00135

**Published:** 2013-07-16

**Authors:** Elisa Ferrando-May, Martin Tomas, Philipp Blumhardt, Martin Stöckl, Matthias Fuchs, Alfred Leitenstorfer

**Affiliations:** ^1^Department of Biology, Bioimaging Center and Center for Applied Photonics, University of KonstanzKonstanz, Germany; ^2^Department of Physics, Chair for Ultrafast Phenomena and Photonics and Center for Applied Photonics, University of KonstanzKonstanz, Germany; ^3^Soft Matter Theory Group, Department of Physics, University of KonstanzKonstanz, Germany

**Keywords:** non-linear optics, fluorescence, microirradiation, DNA strand break

## Abstract

Our understanding of the mechanisms governing the response to DNA damage in higher eucaryotes crucially depends on our ability to dissect the temporal and spatial organization of the cellular machinery responsible for maintaining genomic integrity. To achieve this goal, we need experimental tools to inflict DNA lesions with high spatial precision at pre-defined locations, and to visualize the ensuing reactions with adequate temporal resolution. Near-infrared femtosecond laser pulses focused through high-aperture objective lenses of advanced scanning microscopes offer the advantage of inducing DNA damage in a 3D-confined volume of subnuclear dimensions. This high spatial resolution results from the highly non-linear nature of the excitation process. Here we review recent progress based on the increasing availability of widely tunable and user-friendly technology of ultrafast lasers in the near infrared. We present a critical evaluation of this approach for DNA microdamage as compared to the currently prevalent use of UV or VIS laser irradiation, the latter in combination with photosensitizers. Current and future applications in the field of DNA repair and DNA-damage dependent chromatin dynamics are outlined. Finally, we discuss the requirement for proper simulation and quantitative modeling. We focus in particular on approaches to measure the effect of DNA damage on the mobility of nuclear proteins and consider the pros and cons of frequently used analysis models for FRAP and photoactivation and their applicability to non-linear photoperturbation experiments.

## Introduction

The DNA damage response plays a crucial role in oncogenesis (Bartek et al., 2007, 2012). Consequently, cancer biologists have a strong interest in experimental methods that address the spatiotemporal dynamics of the complex chain of cellular events triggered by DNA lesions. In recent years, microirradiation with focused laser beams has emerged as a useful tool to introduce DNA damage in live cells and to study the ensuing cellular responses via fluorescence imaging. In the first part of this Review, we will recapitulate the pros and cons of the most widespread microirradiation methods and point at new developments involving the use of pulsed near infrared lasers.

In eucaryotes, all reactions to DNA damage occur within chromatin. The local chromatin environment affects the susceptibility to genotoxic agents, influences the choice of DNA repair pathway and the efficiency of the repair reaction (Ziv et al., 2006; Fernandez-Capetillo and Murga, 2008; Jakob et al., 2011; Xu and Price, 2011). Despite successful restoration of the DNA sequence, long-lasting and heritable marks that persist at the level of chromatin structure may contribute to cellular transformation and cancer at a later stage (Lukas et al., 2011). To understand the biological consequences of genotoxic stress it is therefore essential to integrate the role of chromatin into our picture of the DNA damage response. Methods for visualizing chromatin rearrangements induced by DNA damage in living cells at high spatial and temporal resolution are of fundamental importance to achieve this goal. We will present imaging techniques suited to this purpose in the second part of this article focusing on combinations of laser microirradiation and fluorescence photoperturbation. Finally, to achieve an accurate quantitative description of how chromatin dynamics is affected by DNA lesions kinetic data need to be correctly interpreted. We will conclude this Review discussing current modeling approaches and their appropriateness for quantifying alterations of protein mobilities in microirradiated nuclei.

## Laser microirradiation methods for the induction of localized DNA damage

Among the available methods to expose cells to DNA damage like treatment with genotoxic chemicals or diffused irradiation, focused laser beams offer the fundamental advantage of free choice of the target area. With respect to irradiation with heavy ions or alpha particles which can also be employed for this purpose (Heiss et al., 2006), laser sources are easier to handle and to integrate into confocal microscopes for sample observation. Using high aperture objective lenses, the microbeam can be directed to any subcellular or subnuclear region of interest. UV lasers have been used for this purpose since the late 60's and were applied to the study of chromosome structure and of the repair of UV-photolesions in the nucleus (Berns et al., 1969; Berns, 1978; Cremer et al., 1981). In this wavelength region damage to DNA occurs mainly via direct linear absorption. Selective induction of UV-photoproducts, i.e., cyclobutane pyrimidine dimers (CPDs) was reported at 266 nm, a wavelength well-matching the absorption maximum of DNA bases at 260 nm (Voet et al., 1963). At longer wavelengths (340–500 nm), side reactions are observed due to the production of reactive oxygen species in the aqueous cellular environment which also contains endogenous sensitizers. The outcome are (unwanted) oxidative base modifications such as 8-oxo-guanine and DNA strand breaks (Kielbassa et al., 1997). The main microirradiation studies exploiting linear absorption in the UV/VIS region to induce localized DNA damage are summarized in Table 1.

**Table 1:** **Laser microirradiation methods to induce DNA damage predominantly via linear absorption**.

**Reference**	**Laser type**	**Wavelength (nm)**	**Type of DNA damage detected**
Dinant et al., 2007	Diode pumped solid state laser (2 mW, 7.8 kHz)	266	CPD (IF) 6-4PP (IF) no DSB (γH2AX-IF; TUNEL)
Kong et al., 2009	Nitrogen laser (4 ns; 6 Hz, 0.04 μJ/pulse)	337	CPD (IF) 4-6PP (IF) 8-oxoG (IF) DSB (Ku70 recruitment)
Lan et al., 2004	Not reported	365	DSB (γH2AX-IF) 8-oxoG (OGG1 recruitment)
Solarczyk et al., 2012	CW Ar^+^-ion laser (1.7 mW)	488	DSB (γH2AX-IF) DSB (phospho-ATM, RPA, XRCC1, Lig III, PCNA recruitment)

The induced lesions were characterized via immunofluorescence (IF), terminal deoxynucleotidyl transferase dUTP nick end labeling (TUNEL) and recruitment of DNA repair factors, as indicated. CPD, cyclobutane pyrimidine dimers; 6-4PP, 6-4 photoproduct; DSB, DNA strand break; 8-oxoG, 8-oxo-guanine; γH2AX, phosphorylated histone H2AX.

In the presence of exogenous photosensitizers such as the base analogue bromodeoxyuridine (BrdU) or the intercalating dye Hoechst, exposure of cells to UVA illumination leads to single and double strand breaks (Limoli and Ward, 1993). Rougakou et al. exploited this finding to introduce DNA strand breaks in nuclei irradiated with a UVA laser at a wavelength of 390 nm (Rogakou et al., 1999). Since then, the combination of photosensitization and UVA has become very popular because it can be easily performed using a GaN-based UV laser diode at a wavelength of 405 nm which is frequently provided with commercial confocal microscopes. However, at this longer wavelength and only in presence of Hoechst 33342 a residual production of CPD has been reported (see Table 2). The combination of this sensitizer with irradiation at 405 nm seems to trigger an unusual and yet poorly characterized response (Dinant et al., 2007). In general, photosensitizers may have undesired effects on chromatin structure and cellular metabolism, not to mention the fact that they will mediate additional damage throughout the imaging procedure performed with visible light (Solarczyk et al., 2012).

**Table 2:** **Comparison of laser microirradiation methods to induce DNA damage in the presence of photosensitizers**.

**Reference**	**Sensitizer/laser type**	**Wavelength (nm)**	**Type of damage detected**
Lukas et al., 2003	BrdU/nitrogen laser (30 Hz)	337	DSB (γH2AX-IF)
Kong et al., 2009	BrdU/nitrogen laser (4 ns; 6 Hz)	337	DSB (γH2AX-IF)
Rogakou et al., 1999	Hoechst 33258/laser type not reported	390	DSB (γH2AX-IF)
Paull et al., 2000	Hoechst 33258/laser type not reported	390	DSB (γH2AX-IF) 8-oxoG (OGG1)
Kong et al., 2009	BrdU/laser diode (cw)	405	DSB (γH2AX-IF)
Dinant et al., 2007	Hoechst 33342/laser diode (cw)	405	DSB (γH2AX-IF, TUNEL) CPD (IF)
Lan et al., 2005	Hoechst 33342/laser diode (cw)	405	DSB (γH2AX-IF) 8-oxoG (OGG1)
Zarebski et al., 2009	Ethidium bromide/Ar^+^-ion laser (cw)	514	DSB (γH2AX-IF)
			8-oxoG (IF)
			XRCC1 (IF)
			HP1β, HP1γ (IF)
			HP1β, HP1α recruitment

The induced lesions were characterized via immunofluorescence (IF), terminal deoxynucleotidyl transferase dUTP nick end labeling (TUNEL) and recruitment of DNA repair factors, as indicated. CPD, cyclobutane pyrimidine dimer; DSB, DNA strand break; 8-oxoG, 8-oxo-guanine; γH2AX, phosphorylated histone H2AX.

In sum, methods based on linear absorption achieve optimal damage specificity with high efficiency only in the UVC region around 260 nm, leading to almost exclusive induction of UV-photoproducts. This technique requires specialized optics with high UV transmittance. In addition, Solarczyk et al. demonstrated selective induction of DNA strand breaks at 488 nm at power levels normally used in confocal imaging (Solarczyk et al., 2012). Independently of the wavelength used, linear absorption occurs throughout the entire irradiation path. Although the photon flux above and below the focal plane is much lower than in the focus of the objective lens, this may lead to substantial damage and, in flat cultured cells, cause harm to the nuclear membrane.

To achieve confinement of the photomanipulated volume in three dimensions, multiphoton (non-linear) excitation is the tool of choice. First described in theory by Maria Göppert-Mayer in 1931 and demonstrated experimentally by Kaiser and Garrett in 1961, this process relies on the simultaneous absorption of multiple photons at very high photon densities, as they are present within the focus of the objective lens (Göppert-Mayer, 1931; Kaiser and Garrett, 1961). At high numerical apertures the affected volume is restricted to a few femtoliters. The high intensities (GW/cm^2^) required for these transitions are delivered via ultrashort pulses (ps to fs) thus limiting the average laser power to levels compatible with cell viability. For non-linear excitation, the sum of the energy of the incoming photons has to match the definite energy gap between two electronic states. For DNA bases, the maximum of linear absorption lies at 260 nm and excitation at this wavelength leads to the formation of UV-photoproducts, as mentioned above. Hence, the same type of lesion can be generated by irradiating cell nuclei with femtosecond pulses at a wavelength of 780 nm, corresponding to the simultaneous absorption of three near-infrared photons (Meldrum et al., 2003; Trautlein et al., 2008), or in the visible range (~500 nm) via a two-photon process (Daddysman and Fecko, 2011). The relative probabilities of linear and non-linear absorption processes depend strongly on the intensities of the light field applied. Generation of UV-photoproducts via two-photon absorption at the relative moderate intensities used for imaging with continuous wave lasers in the visible range is very inefficient and can thus be neglected.

An additional mechanism that has been proposed to contribute to DNA damage by ultrashort NIR pulses is low-density plasma formation (Kong et al., 2009; Botchway et al., 2010). The high photon density and the extremely strong peak electric field of the laser pulse within the small focal volume may lead to photoionization events. In the presence of water and reactive biomolecules solvated electrons and radical intermediates are formed. This low-density plasma has been observed in pure water after exposure to femtosecond laser pulses over a broad range of conditions (Vogel et al., 2005). Its effects on DNA compare to that of ionizing radiation and include the generation of single and double strand breaks and chemical base modifications.

Thermal heating arising from laser irradiation is expected to play only a minor role as a source of DNA damage. According to calculations of Schönle and Hell irradiation of water for 1 s at a wavelength of 850 nm with 100 mW average power (*NA* = 1.2) causes a temperature rise of 0.2 K due to linear absorption (Schonle and Hell, 1998). Commonly used average powers range far below these conditions. In addition to linear absorption, the high peak power of the ultrashort laser pulses leads to collisions between free electrons and atoms and therefore to a local rise of the temperature. Although this heating can denature biomolecules, the chemical reactions triggered by free electrons strongly dominate the process of DNA damage (Vogel et al., 2005).

So far, most studies have observed a complex mixture of products after non-linear excitation of nuclear DNA (see Table 3). A direct comparison of the results is difficult because the exact irradiation parameters (wavelength, pulse duration, peak power and diameter of the focal spot) are not always fully specified. Furthermore, damage detection methods may vary. Not all types of lesions can be detected directly at the level of the DNA structure. So far, specific and robust antibodies are available for UV-photoproducts only. Methods for the direct detection of base modifications in cells *in situ* are not as well-established. DNA strand breaks have been monitored via TUNEL and Comet assays (Dinant et al., 2007; Harper et al., 2008), but more frequently via antibodies recognizing the phosphorylated form of the histone H2AX (Rogakou et al., 1999), although the latter may also occur subsequent to UV-damage (Halicka et al., 2005). The presence of a defined type of lesion is often inferred from the binding of a fluorescently labeled repair factor in live-cell microscopy experiments. Given the high degree of cross-talk between DNA repair pathways the concomitant occurrence of multiple lesions cannot be excluded by this approach. More extensive reviews comparing the outcome of laser-induced DNA microdamaging methods, including NIR irradiation, are provided in (Dinant et al., 2007; Inagaki et al., 2009; Kong et al., 2009; Nagy and Soutoglou, 2009; Botchway et al., 2010).

**Table 3:** **Laser microirradiation methods to induce DNA damage via non-linear absorption**.

**Reference**	**Wavelength (nm)**	**Laser type**	**Type of damage detected**
Roukos et al., 2011	355	Frequency tripled Nd:YAG laser (470 ps; 500 Hz)	DSB (γH2AX-IF)
Daddysman and Fecko, 2011	400–525	Frequency doubled Ti:sapphire laser (210 fs; 80 MHz)	CPD (IF)
Kong et al., 2009	532	Frequency doubled Nd:YVO4 laser (12 ps; 76 Hz)	CPD (IF) 6-4PP (IF) DSB (Ku70; 53BP1 recruitment) no 8-oxoG (IF)
Meldrum et al., 2003	750	Ti:sapphire laser (120 fs; 82 MHz)	CPD (IF)
Trautlein et al., 2009	775	Frequency doubled Er:fiber laser (230 fs; 107 MHz)	CPD (IF) 6-4PP (IF) DSB (γH2AX-IF)
Mari et al., 2006	800	Ti:sapphire laser (200 fs; 76 MHz)	DSB (γH2AX-IF)
Kong et al., 2009	800	Ti:sapphire laser (200 fs; 76 MHz)	CPD (IF) 6-4PP (IF) DSB (Ku70; 53BP1 recruitment) no 8-oxoG (IF)
Inagaki et al., 2009	800	Ti:sapphire laser (200 fs; 76 MHz)	CPD (IF) DSB (γH2AX-IF)
Dinant et al., 2007	800	Ti:sapphire laser (200 fs; 76 MHz)	CPD (IF) 6-4PP (IF) DSB (γH2AX-IF)
Trautlein et al., 2009	1050	Yb:fiber laser (77 fs; 107 MHz)	no CPD (IF) no 6-4PP (IF) DSB (γH2AX-IF)

The induced lesions were characterized via immunofluorescence (IF), and recruitment of DNA repair factors, as indicated. CPD, cyclobutane pyrimidine dimer; 6-4PP, 6-4 photoproduct; DSB, DNA strand break; 8-oxoG, 8-oxo-guanine; γH2AX, phosphorylated histone H2AX.

Mode locked Ti:sapphire laser oscillators are frequently used to induce DNA damage with NIR irradiation because these systems are well-established as excitation sources for commercial two-photon confocal microscopes. For multiphoton imaging they are usually operated at a wavelength of ~800 nm delivering pulses of a duration of 100–200 fs. From the studies mentioned above it has become clear that these pulses produce a mixture of lesions with strand breaks and photoproducts being detectable at similar levels. What is the perspective for improving the lesion specificity of pulsed NIR irradiation in order to fully exploit the advantages of three-dimensional confinement of the damage and low collateral damage? As we have recently shown, one way to achieve this goal is to increase the center wavelength of the pulse: at λ = 1050 nm the efficiency of formation of UV photoproducts drops drastically while that of DNA strand breaks remains unaltered. This observation let us propose an irradiation method that leads to the preferential induction of DNA strand breaks vs. UV photoproducts without the need of exogenous photosensitizers (Trautlein et al., 2009). As this work suggests, the efficiency of the different damage mechanisms may depend distinctly not only on the wavelength, but also on other pulse parameters. Along this line, Kong et al. have reported an impact of repetition rate and pulse duration on the threshold for phosphorylation of H2AX (Kong et al., 2009) while another study has shown a dependence of cell viability from pulse length at constant pulse energy (Konig et al., 1999).

In sum, non-linear excitation with ultrashort near infrared laser pulses enables to introduce highly localized DNA lesions on a sub-μm scale in the nuclei of living cells with minimum collateral photodamage. As future perspective we envision that these pulses may be tailored to become lesion-specific through a careful analysis of the influence of parameters such as laser central wavelength, pulse duration, repetition rate and peak irradiation on the type of damage.

## Imaging approaches for visualizing chromatin dynamics at sites of local DNA damage

The ability to target only a selected small area of the nucleus via laser microirradiation is one important prerequisite for addressing the role of nuclear organization in the chromatin response to DNA damage. A reliable indicator of chromatin remodeling is histone mobility as can be measured via fluorescence photoperturbation methods. Fluorescence recovery after photobleaching (FRAP), and, to a lesser extent, photoactivation, have yielded important insights into the dynamics and binding properties of histones in intact, native chromatin (Kimura, 2005; Beaudouin et al., 2006; Wiesmeijer et al., 2008; Martin and Cardoso, 2010; Raghuram et al., 2010; Stasevich et al., 2010). Mobility changes triggered by DNA damage can be investigated easily on a global scale, by treating cells with genotoxic agents such as chemicals or ionizing radiation prior to a FRAP or photoactivation experiment. These approaches disregard local differences in chromatin architecture, and they detect mobility changes only on the time-scale of several minutes to hours depending on the type and duration of the genotoxic treatment. To study the effect of local DNA damage on chromatin dynamics one can perform a FRAP experiment within a subnuclear region previously exposed to the DNA damaging laser. This strategy is straightforward if the fluorescently labeled protein of interest visibly binds to the damage and accrues along the laser trajectory. The photobleaching laser is then targeted to this area when the signal increase, i.e., the binding reaction in the irradiated region, has reached steady-state to avoid superimposition of the recruitment and the bleaching recovery kinetics. Using this approach, Mortusevicz et al have shown distinct mobilities of XRCC1 and PCNA at DNA damage induced at 405 nm (Mortusewicz and Leonhardt, 2007), while two other studies have investigated the biphasic dynamics of HP1-β and compared the mobilities of Cdt1, Cdt2 and PCNA at laser-irradiated sites, respectively (Ayoub et al., 2008; Roukos et al., 2011). Further reports compared the recovery kinetics of PARP1 and a catalytic mutant at DNA damage (Mortusewicz et al., 2007). The behavior of members of the MRN complex and of 53BP1 at DNA strand breaks was also investigated (Lukas et al., 2004; Bekker-Jensen et al., 2005). For proteins that do not recruit to DNA damage including most chromatin components it is possible to measure FRAP curves within a pre-irradiated, damaged area via its known coordinates. However, unintentional bleaching due to the damaging laser—in particular if wavelengths in the UV/VIS are used—will interfere with the subsequent FRAP experiment. The latter can be performed only after the signal has recovered from the first irradiation step, thus severely limiting the temporal resolution of this approach.

Fluorescence photoactivation has also been combined with laser microirradiation to study local changes of chromatin structure due to DNA strand breaks (Kruhlak et al., 2006). In this study, cells expressing a photoactivatable fusion of histone H2B (PAGFP-H2B) were irradiated with a UV laser (364 nm) either in the absence or presence of the DNA intercalator Hoechst 33342. DNA strand breaks were produced only under the latter condition, as demonstrated by the appearance of phosphorylated H2A along the laser track. Under both conditions the DNA damaging laser simultaneously activated PAGFP-H2B. Significantly, a local broadening of the fluorescence signal could be observed only in the presence of the sensitizer indicating an expansion of chromatin in the direct proximity of DNA strand breaks.

These examples highlight one basic requirement for combining DNA damage via laser microirradiation with fluorescence photoperturbation for mobility measurements: the two irradiation procedures must not influence each other, i.e., they have to either induce DNA damage or photobleach/photoactivate, respectively. In the study by Kruhlak et al., this distinction was reached by adding a photosensitizer. Recently, we have presented an alternative strategy based on the use of femtosecond laser pulses of different wavelengths (Tomas et al., 2012). In a first step, DNA strand breaks are introduced via irradiation at λ = 1050 nm in a defined subnuclear volume, as described above. Subsequently the protein to be studied, which is expressed as a photoactivatable fusion is spot-activated within the lesioned zone via two-photon absorption at λ = 775 nm. We demonstrate that the two irradiation procedures can be performed in a single cell nucleus without mutual interference if the pulse parameters are chosen properly. Under these conditions it is possible to vary the time delay between the infliction of damage at 1050 nm and the photoactivation step at 775 nm arbitrarily. Hence, alterations in protein mobilities can be probed at different stages of the DNA damage response. The spatial precision of non-linear excitation also enables to vary the position of the photoactivation spot with respect to the damaged region. Using this method, we could show that DNA strand breaks lead to an increase in the mobility of histone H1.2 in the time range of 2 min after infliction of damage and that this change is spatially confined because distant chromatin is not affected (Tomas et al., 2012). The spatial and temporal flexibility of this assay will enable to visualize how the chromatin response to DNA damage emanates within the cell nucleus thus contributing to dissect the spatiotemporal complexity of this fundamental biological process.

## Modeling changes in chromatin dynamics in response to local DNA damage

To extract information from the complex data sets on the kinetics generated by the combination of laser microdamage and fluorescence photoperturbation it is necessary to elaborate proper modeling approaches. Their aim is to describe quantitatively how structural damage affects the interaction of DNA with chromatin proteins.

Numerous studies have undertaken quantitative analysis of FRAP and photoactivation data of nuclear proteins under undisturbed conditions (i.e., in the absence of intentional DNA damage). In general, a reaction-diffusion model is assumed to be the best mathematical description of the dynamic behavior of these proteins. According to our current understanding, nuclear proteins diffuse stochastically in all three dimensions within the nucleoplasm until they collide with binding partners with whom they undergo transient interactions. This model assigns to each protein the following characteristic parameters: the binding and release constants *k*_on_ and *k*_off_ describing binding events, and the diffusion coefficient D accounting for phases of unrestrained movement. Solving the system of differential equations required for a full reaction-diffusion model is a complex task. Therefore, different simplifying assumptions have been made in different studies, depending on the experimental setting used to generate the mobility data (FRAP, or photoactivation, size of the bleached/photoactivated area etc.). These simplifications have enabled analytical solutions of the reaction-diffusion model, exactly describing the redistribution of the fluorescence signal to equilibrium as a function of time. The basic assumptions that are employed for this type of approach are the following:

1. The system's properties remain close to unperturbed, i.e., the number and the distribution of binding sites do not change during the observation period. In addition, binding sites are assumed to be homogenously distributed within the nucleus. The latter does not hold true for chromatin proteins because chromatin density varies between subnuclear regions (e.g., in hetero- vs. euchromatin.).
2. The photobleached/photoactivated volume has a uniform extension along the z-dimension. According to this assumption intensity changes introduced by the photobleaching/-activating laser do not vary along the optical axis, and the system can be described by a two-dimensional model.
3. The finite dimension and the geometry of the cell nucleus can be neglected. This simplification applies only when the photobleached/photoactivated spot is small as compared to the nuclear volume.

Otherwise, the solution of the reaction-diffusion model has to be found via numerical modeling where changes in fluorescence are approximated consecutively for each time point for a given set of parameters. For both the analytical and the numerical procedure, the obtained solutions are optimized by testing combinations of different parameters iteratively until the simulated behavior fits the experimental data.

The power of kinetic modeling consists in its ability to make quantitative predictions about the reaction of the biological system under study. For FRAP models, this ability has been questioned, because different approaches have yielded very different results for the same or similar proteins. Therefore, a cross-validation strategy that compares different models as well as different experimental methods to generate the primary data is highly recommended (Mazza et al., 2008, 2012; Mueller et al., 2010).

For modeling changes of nuclear protein dynamics triggered by localized DNA damage yet other issues have to be considered. The main difference with respect to the undamaged situation is that at the time point of the mobility measurement the system itself may not be in an unperturbed state (see point 1 in the above list). Exposure to short laser pulses to induce DNA damage initiates a signal chain that may develop on different time scales for different types of lesions/binding sites. This amplification process is inherent to the biological response to DNA damage and leads to a fast and massive propagation of the damage signal, as best exemplified by the spreading of the phosphorylation of histone H2AX from the initial strand break to chromatin regions of the size of a few megabases (Rogakou et al., 1999; Iacovoni et al., 2010). Docking sites for proteins are thus generated in reactions secondary to proper DNA damage, as has been described in detail for many DNA repair factors, but also holds true for chromatin constituents (see e.g., Li et al., 2010; Lukas et al., 2011; Xu et al., 2012). To verify if the steady-state criterium is fulfilled it is thus very important to assess qualitatively on which time scale the mobility of the protein under investigation is influenced by DNA damage. The equilibrium approximation can be made only for observation times that are much shorter than the chromatin remodeling processes themselves.

Concerning the number and distribution of binding sites, a spatial discontinuity is implicit in the localized nature of the damage inflicted by microirradiation. Obviously, a different number of binding sites is present within the damaged region as compared to the rest of the nucleoplasm. The approximation of a uniform distribution can be made only if the area in which fluorescence redistribution is measured is much smaller than the DNA damaged one, and only for short observation times during which the protein does not move outside of this region. In our assay, this condition is fulfilled since the protein of interest is photoactivated in a small spot (diameter 1 μm) within a significantly larger zone containing the damage (6 μm^2^) (Tomas et al., 2012). These caveats concerning the temporal and spatial equilibrium criteria apply in general to all methods combining microirradiation with fluorescence photoperturbation.

An aspect that needs to be specifically considered when attempting to model data from non-linear photoactivation experiments concerns the shape of the photoactivated volume. Due to the three-dimensional confinement of non-linear excitation the signal intensity is not uniform along the optical axis. Thus, the conditions for a two-dimensional approximation are not fulfilled strictly (point 2). On the other hand, the small spot size minimizes the influence of nuclear geometry which may therefore be neglected without inducing significant errors (point 3).

An overview of recently proposed approaches for the analysis of FRAP and photoactivation experiments specifying their most important features is given in Table 4. The study of Mazza et al. addresses the point of the three-dimensionally confined nonlinear excitation volume and proposes an analytical solution of the diffusion equation for a two-photon photoactivated/bleached circular area of variable size. The method enables to determine diffusion coefficients over a wide range of values, but binding, i.e., a reaction dominated protein dynamics, is only taken into account as an immobile fraction. A solution for the reaction-diffusion model is not given (Mazza et al., 2008). Sprague et al. develop an analytical solution of the reaction-diffusion model which applies to a spatially localized cluster of binding sites and use it to simulate binding of the glucocorticoid receptor to an array of promoter sites in live cells. This approach has the advantage of taking into account a non-homogenous distribution of binding sites as it occurs in microirradiated cell nuclei. However, it is only applicable if the size of the cluster (the damaged area) is smaller than the bleached/photoactivated region. This model has been recently applied to describe the behavior of Nbs1 and Mdc1 at sites of DNA damage introduced by charged particle irradiation (Sprague et al., 2006; Tobias et al., 2013).

**Table 4:** **Selection of recently proposed analysis models for FRAP and photoactivation (PA) experiments**.

**Reference**	**Model**	**Solution/dimension**	**Bleached/photoactivated area**	**Remarks**
Sprague et al., 2004	Reaction-diffusion	Analytical; 2D	Circular	Laplace transform of the solution; simplified solutions in explicit form for different values of association and dissociation rates
Carrero et al., 2004	Reaction-diffusion, and compartment model	Analytical; 1D/2D	Line/rectangular	Explicit solution for a bleached stripe; considers biphasic behavior of diffusion and binding
Phair et al., 2004	Reaction dominant, no diffusion	Analytical; 2D	50% of the nucleus	Considers only binding, diffusion is neglected
Sprague et al., 2006	Reaction-diffusion	Analytical; 2D	Circular	Subnuclear compartments with different binding rates in axial direction can be regarded
Mazza et al., 2008	Pure diffusion and immobile fraction	Analytical; 2D/3D	Circular	Appropriate for multiphoton FRAP/PA
Kang and Kenworthy, 2008	Reaction-diffusion	Analytical; 2D	Uniform circle or Gaussian	Explicit solution (no Laplace transform as in Sprague et al.)
Beaudouin et al., 2006	Reaction-diffusion	Numerical; 2D	50% of the nucleus	Considers nuclear geometry and inhomogeneous distribution of binding sites
Calvert et al., 2007	Diffusion	Numerical; 3D	Gaussian ellipsoid	Multiphoton excitation, spherical boundary condition, model calculates intensity along a line
Stasevich et al., 2010	Reaction-diffusion	Numerical; 2D	Circular	Model calculates intensity along a line across the bleach profile

Numerical solutions of the reaction-diffusion model provide a more accurate approach to describe protein dynamics. In their study, Beaudouin et al. present a method that includes both the real geometry of the nucleus and an inhomogeneous distribution of binding sites and is independent of the shape of the bleached/photoactivated region. The differential equations are solved numerically using a finite difference method. The approach was validated for the photoactivation of five different chromatin proteins (Beaudouin et al., 2006). It is interesting to note that in the case of histone H1, these authors do not detect subpopulations with higher and lower mobility, contrarily to what was reported previously in FRAP studies (Raghuram et al., 2010; Stasevich et al., 2010). Since the bleaching laser may induce phototoxic effects including DNA damage (Dobrucki et al., 2007; Solarczyk et al., 2012) and the mobility of the protein is increased in the presence of such lesions (Tomas et al., 2012), the partial fluorescence recovery observed in FRAP experiments can be due to a change of the protein's mobility within the bleached area leading to a local decrease in concentration rather than to the presence of an immobile fraction. These effects have to be considered when choosing experimental conditions for photoperturbation studies.

None of the currently available models addresses the issue of the temporal non-equilibrium of the system that is characteristic for mobility measurements performed subsequently to DNA microdamage, either via FRAP or photoactivation. Addressing this issue is a promising avenue for future work and a prerequisite for the proper quantitative description of the response of nuclear proteins to DNA damage.

### Conflict of interest statement

The authors declare that the research was conducted in the absence of any commercial or financial relationships that could be construed as a potential conflict of interest.
